# A Dual Measure of Uncertainty: The Deng Extropy

**DOI:** 10.3390/e22050582

**Published:** 2020-05-21

**Authors:** Francesco Buono, Maria Longobardi

**Affiliations:** 1 Dipartimento di Matematica e Applicazioni “Renato Caccioppoli”, Università degli Studi di Napoli Federico II, I-80126 Naples, Italy; francesco.buono3@unina.it; 2 Dipartimento di Biologia, Università degli Studi di Napoli Federico II, I-80126 Naples, Italy

**Keywords:** the Dempster–Shafer evidence theory, Deng entropy, extropy, pattern recognition

## Abstract

The extropy has recently been introduced as the dual concept of entropy. Moreover, in the context of the Dempster–Shafer evidence theory, Deng studied a new measure of discrimination, named the Deng entropy. In this paper, we define the Deng extropy and study its relation with Deng entropy, and examples are proposed in order to compare them. The behaviour of Deng extropy is studied under changes of focal elements. A characterization result is given for the maximum Deng extropy and, finally, a numerical example in pattern recognition is discussed in order to highlight the relevance of the new measure.

## 1. Introduction

Let *X* be a discrete random variable with support $\left\{x_{1},\ldots ,x_{n}\right\}$ and with probability mass function vector $\underline{p}=\left(p_{1},\ldots ,p_{n}\right)$. The Shannon entropy of *X* is defined as

(1) $$H\left(X\right)=H\left(\underline{p}\right)=-\sum_{i=1}^{n}p_{i}\log p_{i},$$

where log is the natural logarithm, see [1]. It is a measure of information and discrimination about the uncertainty related to the random variable *X*. Lad et al. [2] proposed the extropy as the dual of entropy. This is a measure of uncertainty related to the outside and is defined as

(2) $$J\left(X\right)=J\left(\underline{p}\right)=-\sum_{i=1}^{n}\left(1-p_{i}\right)\log\left(1-p_{i}\right).$$

Lad et al. proved the following property related to the sum of the entropy and the extropy:

(3) $$H\left(\underline{p}\right)+J\left(\underline{p}\right)=\sum_{i=1}^{n}H\left(p_{i},1-p_{i}\right)=\sum_{i=1}^{n}J\left(p_{i},1-p_{i}\right),$$

where $H\left(p_{i},1-p_{i}\right)=J\left(p_{i},1-p_{i}\right)=-p_{i}\log p_{i}-\left(1-p_{i}\right)\log\left(1-p_{i}\right)$ are the entropy and the extropy of a discrete random variable of which the support has cardinality two and the probability mass function vector is $\left(p_{i},1-p_{i}\right)$.

Dempster [3] and Shafer [4] introduced a method to study uncertainty. Their theory of evidence is a generalization of the classical probability theory. In D-S theory, an uncertain event with a finite number of alternatives is considered, and a mass function over the power set of the alternatives (i.e., a degree of confidence to all of its subsets) is defined. If we give positive mass only to singletons, a probability mass function is obtained. D-S theory allows us to describe more general situations in which there is less specific information.

Here we describe an example studied in [5] to explain how D-S theory extends the classical probability theory. Consider two boxes, *A* and *B* such that in *A* there are only red balls, whereas in *B* there are only green balls and the number of balls in each box is unknown. A ball is picked randomly from one of these two boxes. The box *A* is selected with probability $p_{A}=0.6$ and box *B* is selected with probability $p_{B}=0.4$. Hence, the probability of picking a red ball is 0.6, P(R)=0.6, whereas the probability of picking a green ball is 0.4, P(G)=0.4. Suppose now that in box *B* there are green and red balls with rates unknown. The box *A* is still selected with probability $p_{A}=0.6$ and the box *B* with probability $p_{B}=0.4$. In this case, we can not obtain the probability of picking a red ball. To analyze this problem, we can use D-S theory to express the uncertainty. In particular, we choose a mass function *m* such that, m(R)=0.6 and m(R,G)=0.4.

Dempster–Shafer theory of evidence has several applications due to its advantages in dealing with uncertainty; for example, it is used in decision making [6,7], in risk evaluation [8,9], in reliability analysis [10,11] and so on. In the following, we recall the basic notions of this theory.

Let *X* be a frame of discernment, i.e., a set of mutually exclusive and collectively exhaustive events indicated by $X=\{\theta_{1},\theta_{2},\ldots ,\theta_{\left|X\right|}\}$. The power set of *X* is indicated by $2^{X}$ and it has cardinality $2^{\left|X\right|}$. A function $m:2^{X}\to \left[0,1\right]$ is called a mass function or a basic probability assignment (BPA) if

$$m\left(\emptyset \right)=0\;\mathrm{and}\;\sum_{A\in 2^{X}}m\left(A\right)=1.$$

If m(A)≠0 implies |A|=1 then *m* is also a probability mass function, i.e., BPAs generalize discrete random variables. Moreover, elements *A* such that m(A)>0 are called focal elements. Given a BPA, we can evaluate for each focal element the pignistic probability transformation (PPT). Let us recall that the pignistic probability is the probability that a thinking being would assign to that event. It represents a point estimate of belief and can be determined as [12]

(4) $$PPT\left(A\right)=\sum_{B:A\subseteq B}\frac{m(B)}{\left|B\right|}.$$

If we have a weight or a reliability of evidence, represented by a coefficient α∈[0,1], we can use it to generate another BPA $m^{\alpha }$ in the following way (see [4])

(5) $$m^{\alpha }\left(A\right)=\left\{\begin{array}{cc} \alpha \;m(A), & \mathrm{if}A\subset X\\ \alpha \;m(X)+(1-\alpha ), & \mathrm{if}A=X. \end{array}\right.$$

If we have two BPAs $m_{1},\;m_{2}$ for a frame of discernment *X*, we can introduce another BPA $m^{*}$ for *X* using the Dempster rule of combination, see [3]. We define $m^{*}\left(A\right)$, A⊆X, in the following way

(6) $$m^{*}\left(A\right)=\left\{\begin{array}{cc} 0, & \mathrm{if}A=\emptyset \\ \frac{\sum_{B,C\subseteq X:B\cap C=A}m_{1}\left(B\right)m_{2}\left(C\right)}{1-K}, & \mathrm{if}A\neq \emptyset \end{array}\right.$$

where $K=\sum_{B,C\subseteq X:B\cap C=\emptyset }m_{1}\left(B\right)m_{2}\left(C\right)$. We remark that, if K>1, we can not apply the Dempster rule of combination.

Recently, several measures of discrimination and uncertainty have been proposed in the literature (see, for instance, [13,14,15,16,17,18,19]). In particular, in the context of the Dempster–Shafer evidence theory, there are interesting measures of discrimination, as the Deng entropy; it was this latter concept that has suggested us the introduction of a dual definition.

The Deng entropy was introduced in [5] for a BPA *m* as

(7) $$E_{d}\left(m\right)=-\sum_{A\subseteq X:m(A)>0}m\left(A\right)\log_{2}\left(\frac{m(A)}{2^{\left|A\right|}-1}\right).$$

This entropy is similar to Shannon entropy and they coincide if the BPA is also a probability mass function. The term $2^{\left|A\right|}-1$ represents the potential number of states in *A*. For a fixed value of m(A), as the cardinality of *A* increases, $2^{\left|A\right|}-1$ increases and then also Deng entropy does.

In the literature, several properties of Deng entropy have been studied (see for instance [20]) and other measures of uncertainty based on Deng entropy have been introduced (see [21,22]). Other relevant measures of uncertainty and information known in the Dempster–Shafer theory of evidence are, for example, Hohle’s confusion measure [23], Yager’s dissonance measure [24] and Klir and Ramer’s discord measure [25].

The aim of this paper is to dualize the Deng entropy by defining a corresponding extropy. We present some examples of comparing Deng entropy and our new extropy and their monotonicities. Then investigate the relations between these measures, and the behaviour of the Deng extropy under changes of focal elements. Moreover, a characterization result is given for the maximum Deng extropy. Finally, an application to pattern recognition is given in which it is clear that the Deng extropy can make the right recognition. In the conclusions, the results contained in the paper are summarized.

## 2. The Deng Extropy

In order to obtain an analogue of Equation (3), we choose the following definition for the Deng extropy

(8) $$EX_{d}\left(m\right)=-\sum_{A\subset X:m(A)>0}\left(1-m\left(A\right)\right)\log_{2}\left(\frac{1-m(A)}{2^{|A^{c}|}-1}\right),$$

where $A^{c}$ is the complementary of *A* in *X* and $|A^{c}\left|=|X|-|A\right|$. Our purpose is to apply the Deng extropy in order to measure the uncertainty related to the outside in the context of Dempster–Shafer evidence theory. For this reason, *X* is not involved in the determination of the Deng extropy, even when m(X)>0. The term $2^{|A^{c}|}-1$ represents the potential number of states outside of *A*. For a fixed value of m(A), as the cardinality of *A* increases, $2^{|A^{c}|}-1$ decreases and then also the Deng extropy does.

**Proposition** **1.** *Let m be a BPA for a frame of discernment X. Then*

(9) $$\begin{array}{ccc} E_{d}\left(m\right)+EX_{d}\left(m\right) & = & \sum_{A\subset X:m(A)>0}E_{d}\left(m_{A}^{*}\right)-m\left(X\right)\log_{2}\left(\frac{m(X)}{2^{\left|X\right|}-1}\right) \end{array}$$

(10) $$\begin{array}{ccc} & = & \sum_{A\subset X:m(A)>0}EX_{d}\left(m_{A}^{*}\right)-m\left(X\right)\log_{2}\left(\frac{m(X)}{2^{\left|X\right|}-1}\right), \end{array}$$

*with the convention 0log0=0, where $m_{A}^{*}$ is a BPA on X defined as*

$$m_{A}^{*}\left(B\right)=\left\{\begin{array}{cc} m(A), & if\;B=A\\ 1-m(A), & if\;B=A^{c}\\ 0, & otherwise. \end{array}\right.$$

**Proof.**  From the definition of the BPA $m_{A}^{*}$ we have

$$\begin{array}{cc} E_{d}\left(m_{A}^{*}\right) & =-m\left(A\right)\log_{2}\frac{m(A)}{2^{\left|A\right|}-1}-\left(1-m\left(A\right)\right)\log_{2}\frac{1-m(A)}{2^{|A^{c}|}-1}\\ EX_{d}\left(m_{A}^{*}\right) & =-\left(1-m\left(A\right)\right)\log_{2}\frac{1-m(A)}{2^{|A^{c}|}-1}-m\left(A\right)\log_{2}\frac{m(A)}{2^{\left|A\right|}-1}, \end{array}$$

i.e., they are equal. Hence, for every A⊂X such that m(A)>0 $E_{d}\left(m_{A}^{*}\right)$ (or $EX_{d}\left(m_{A}^{*}\right)$) gives the corresponding addend of $E_{d}\left(m\right)+EX_{d}\left(m\right)$. The only exception is given by *X*, which could give a contribution in the left hand side of Equation (9) if m(X)>0, and for this reason we have the extra term in the right side of Equations (9) and (10). □

Next, we give some examples of evaluation of the Deng extropy and entropy in different situations.

**Example** **1.** *Given a frame of discernment X, a∈X and a BPA m such that m({a})=m(a)=1, we have*

$$\begin{array}{cc} \; & EX_{d}\left(m\right)=-\left(1-1\right)\log_{2}\frac{1-1}{2^{|X|-1}-1}=0,\\ \; & E_{d}\left(m\right)=-\log_{2}1=0. \end{array}$$

*So, in this case, Deng entropy coincides with Deng extropy and they are equal to* 0.

**Example** **2.** *Given a frame of discernment X={a,b,c} and a BPA m such that $m\left(a\right)=m\left(b\right)=m\left(c\right)=\frac{1}{3}$, we have*

$$\begin{array}{cc} \; & EX_{d}\left(m\right)=-\frac{2}{3}\log_{2}\left(\frac{2}{9}\right)\cdot 3=-2\log_{2}\frac{2}{9},\\ \; & E_{d}\left(m\right)=-\frac{1}{3}\log_{2}\left(\frac{1}{3}\right)\cdot 3=-\log_{2}\frac{1}{3}. \end{array}$$

**Example** **3.** *Given a frame of discernment X={a,b,c} and a BPA m such that $m\left(a\right)=m\left(b\right)=m\left(c\right)=m\left(a,b\right)=\frac{1}{4}$, we have*

$$\begin{array}{cc} \; & EX_{d}\left(m\right)=-\frac{3}{4}\log_{2}\left(\frac{1}{4}\right)\cdot 3-\frac{3}{4}\log_{2}\frac{3}{4}=3-\frac{3}{4}\log_{2}3,\\ \; & E_{d}\left(m\right)=-\frac{1}{4}\log_{2}\left(\frac{1}{4}\right)\cdot 3-\frac{1}{4}\log_{2}\frac{1}{12}=2+\frac{1}{4}\log_{2}3. \end{array}$$

**Example** **4.** *Given a frame of discernment X with cardinality n and a BPA m such that $m\left(i\right)=\frac{1}{n}$, for i=1,…,n, we have*

$$\begin{array}{cc} EX_{d}\left(m\right) & =-n\left(1-\frac{1}{n}\right)\log_{2}\left(\frac{1-\frac{1}{n}}{2^{n-1}-1}\right)\\ & =\left(n-1\right)\left[\log_{2}\left(\frac{n}{n-1}\right)+\log_{2}\left(2^{n-1}-1\right)\right],\\ E_{d}\left(m\right) & =\log_{2}\left(n\right), \end{array}$$

**Figure 1:**
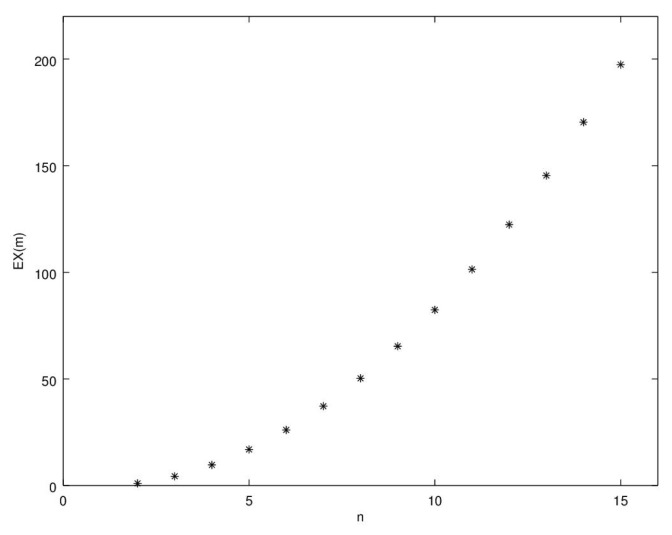
$EX_{d}\left(m\right)$ in function of *n* with basic probability assignment (BPA) defined in Example 4.

*which are increasing with n∈N. We plot the values of the Deng extropy for n≤15 in Figure 1. Deng entropy is not plotted because it is trivial.*

**Example** **5.** *Given a frame of discernment X={1,2,…,15} and a BPA m such that m(3,4,5)=0.05, m(6)=0.05, m*(*A*) = 0.8, m(X)=0.1. *When A changes, the following values for the Deng extropy and entropy in Table 1 are obtained:*

*As it was pointed out before, the results show that the extropy of m decreases monotonously with the rise of the size of subset A, while the entropy increases.*

## 3. The Maximum Deng Extropy

Kang and Deng [26] studied the problem of the maximum Deng entropy. They find out that the maximum Deng entropy on a frame of discernment *X* with cardinality |X| is attained if and only if the BPA *m* is defined as

$$m\left(A_{i}\right)=\frac{2^{|A_{i}|}-1}{\sum_{j=1}^{2^{\left|X\right|}-1}\left(2^{|A_{j}|}-1\right)},\;\;\;i=1,\ldots ,2^{\left|X\right|}-1,$$

where $A_{i}$, $i=1,\ldots ,2^{\left|X\right|}-1$, are all non-empty elements of $2^{X}$. Hence, the value of the maximum Deng entropy is given by

$$E_{d}^{*}=-\sum_{i=1}^{2^{\left|X\right|}-1}\frac{2^{|A_{i}|}-1}{\sum_{j=1}^{2^{\left|X\right|}-1}\left(2^{|A_{j}|}-1\right)}\log_{2}\frac{\frac{2^{|A_{i}|}-1}{\sum_{j=1}^{2^{\left|X\right|}-1}\left(2^{|A_{j}|}-1\right)}}{2^{|A_{i}|}-1}=\log_{2}\sum_{i=1}^{2^{\left|X\right|}-1}\left(2^{|A_{i}|}-1\right).$$

In this section, we provide conditions to obtain the maximum Deng extropy for a fixed number of focal elements and with a fixed value for m(X).

**Theorem** **1.** *Let m be a BPA for a frame of discernment X. The maximum Deng extropy for fixed values of m(X) and N number of focal elements different from X, N=|N={A⊂X:m(A)>0}, is attained if and only if*

$$m\left(A\right)=1-\frac{N-(1-m(X))}{\sum_{B\in N}\left(2^{|B^{c}|}-1\right)}\left(2^{|A^{c}|}-1\right),\;\;\;A\in N.$$

*In this case, the value of the maximum Deng extropy is*

$$EX_{d}^{*}=-\left[N-\left(1-m\left(X\right)\right)\right]\log_{2}\frac{N-(1-m(X))}{\sum_{A\in N}\left(2^{|A^{c}|}-1\right)}.$$

**Proof.**  Suppose m(X)=0. We will prove that in this case the maximum Deng extropy is

$$EX_{d}^{*}=-\left(N-1\right)\log_{2}\frac{N-1}{\sum_{A\in N}\left(2^{|A^{c}|}-1\right)},$$

and that it is attained if and only if the BPA is defined by

(11) $$m\left(A\right)=1-\frac{N-1}{\sum_{B\in N}\left(2^{|B^{c}|}-1\right)}\left(2^{|A^{c}|}-1\right),\;\;\;A\in N.$$

We have to maximize

$$EX_{d}=-\sum_{A\in N}\left(1-m\left(A\right)\right)\log_{2}\frac{1-m(A)}{2^{|A^{c}|}-1}$$

subject to the condition

$$\sum_{A\in N}m\left(A\right)=1.$$

Then, the Lagrange function can be defined as

$${\left(EX_{d}\right)}_{0}=-\sum_{A\in N}\left(1-m\left(A\right)\right)\log_{2}\frac{1-m(A)}{2^{|A^{c}|}-1}+\lambda \left(\sum_{A\in N}m\left(A\right)-1\right).$$

Thus the gradient can be computed, and for A∈N we have

$$\frac{\partial {\left(EX_{d}\right)}_{0}}{\partial m(A)}=\log_{2}\frac{1-m(A)}{2^{|A^{c}|}-1}+\log_{2}e+\lambda =0,$$

where $\log_{2}e+\lambda$ does not depend on m(A). By vanishing all the partial derivatives, we obtain

$$\frac{1-m(A)}{2^{|A^{c}|}-1}=K,\;\;\;A\in N,$$

where *K* is a constant. It follows

(12) $$m\left(A\right)=1-K\left(2^{|A^{c}|}-1\right),\;\;\;A\in N.$$

By summing over A∈N, we get

$$1=\sum_{A\in N}\left(1-K\left(2^{|A^{c}|}-1\right)\right),$$

and then

$$K=\frac{N-1}{\sum_{A\in N}\left(2^{|A^{c}|}-1\right)}.$$

Therefore, from Equation (12) we obtain

$$m\left(A\right)=1-\frac{N-1}{\sum_{B\in N}\left(2^{|B^{c}|}-1\right)}\left(2^{|A^{c}|}-1\right),\;\;\;A\in N,$$

i.e., Equation (11). Finally, for the Deng extropy related to this BPA, we get

$$\begin{array}{cc} EX_{d}\left(m\right) & =-\sum_{A\in N}\frac{N-1}{\sum_{B\in N}\left(2^{|B^{c}|}-1\right)}\left(2^{|A^{c}|}-1\right)\log_{2}\frac{N-1}{\sum_{B\in N}\left(2^{|B^{c}|}-1\right)}\\ & =-\left(N-1\right)\log_{2}\frac{N-1}{\sum_{A\in N}\left(2^{|A^{c}|}-1\right)}=EX_{d}^{*} \end{array}$$

and the proof is completed. □

## 4. Application to Pattern Recognition

In this section, we investigate an application of the Deng extropy in pattern recognition by using the dataset Iris given in [27]. This example was already studied in [28] to analyze the applications of another belief entropy defined in the Dempster–Shafer evidence theory. We compare a method proposed by Kang [29] with a method based on the use of Deng extropy. The Iris dataset is useful to introduce the application of the generation of BPAs based on the Deng extropy in the classification of the kind of flowers. The dataset is composed of 150 samples. For each one, we have the sepal length in cm (SL), the sepal width in cm (SW), the petal length in cm (PL), the petal width in cm (PW) and the class that is only one between Iris Setosa (Se), Iris Versicolour (Ve) and Iris Virginica (Vi). The samples are equally distributed for each class. We select 40 samples for each kind of Iris and then we use sample of max–min value to generate a model of interval numbers, as shown in Table 2. Each element of the dataset can be regarded as an unknown test sample. Suppose the selected sample data is [6.1, 3.0, 4.9, 1.8, Iris Virginica].

Four BPAs are generated with a method proposed by Kang et al. based on the similarity of interval numbers [29]. Given two intervals $A=[a_{1},a_{2}]$ and $B=[b_{1},b_{2}]$ their similarity S(A,b) is defined as

$$S\left(A,B\right)=\frac{1}{1+\alpha \;D(A,B)},$$

where α>0 is the coefficient of support (we choose α=5) and D(A,B) is the distance of intervals *A* and *B* defined in [30] as

$$D^{2}\left(A,B\right)={\left[\left(\frac{a_{1}+a_{2}}{2}\right)-\left(\frac{b_{1}+b_{2}}{2}\right)\right]}^{2}+\frac{1}{3}\left[{\left(\frac{a_{2}-a_{1}}{2}\right)}^{2}+{\left(\frac{b_{2}-b_{1}}{2}\right)}^{2}\right].$$

In order to generate BPAs, the intervals given in Table 2 are used as interval *A* and as interval *B* we use singletons given by the selected sample. For each one of the four properties, we get seven values of similarity and then we get a BPA by normalizing them (see Table 3). Hence, we evaluate the Deng extropy of these BPAs, as shown in the bottom row of Table 3. We obtain a combined BPA by using the Dempster rule of Combination (6). The type of unknown sample is determined by combined BPA. From Equation (4), we get the maximum value of PPT. Hence, Kang’s method assigns to the sample the type Iris Versicolour and it does not make the right decision.

Next, we use the Deng extropies given in Table 3 to generate other BPAs. We refer to these extropies as $EX_{d}\left(SL\right),\;EX_{d}\left(SW\right),\;EX_{d}\left(PL\right),\;EX_{d}\left(PW\right)$. We use these values as significance of each sample property to evaluate the weight of each property. For the sample property sepal length we have

$$\omega \left(SL\right)=\frac{e^{-EX_{d}\left(SL\right)}}{e^{-EX_{d}\left(SL\right)}+e^{-EX_{d}\left(SW\right)}+e^{-EX_{d}\left(PL\right)}+e^{-EX_{d}\left(PW\right)}}.$$

We divide each weight for the maximum of the weights and use these values as discounting coefficients to generate new BPAs, as shown in Equation (5), see Table 4. Again, a combined BPA is obtained by using the Dempster rule of combination. The type of unknown sample is determined by combined BPA. Hence, the method based on the Deng extropy can make the right recognition.

We tested all 150 samples and we get that the global recognition rate of Kang’s method is 93.33% whereas the global recognition of the method based on the Deng extropy is 94%. The results are shown in Table 5.

## 5. Conclusions

In this paper, the Deng extropy has been defined as the dual measure of Deng entropy. Its relation with the analogous entropy has been analyzed and these measures have been compared in order to decide in which cases the first is better than the other one. Moreover, some examples are proposed. The behaviour of Deng extropy has been studied under changes of focal elements. We have given a characterization result for the maximum Deng extropy and, finally, a numerical example is discussed in order to highlight the relevance of this dual measure in pattern recognition.

## Figures and Tables

**Table 1:** The value of the Deng extropy and the Deng entropy when *A* changes.

A	Deng Extropy	Deng Entropy
{1}	28.104	2.6623
{1,2}	27.904	3.9303
{1,2,3}	27.704	4.9082
{1,…,4}	27.504	5.7878
{1,…,5}	27.304	6.6256
{1,…,6}	27.104	7.4441
{1,…,7}	26.903	8.2532
{1,…,8}	26.702	9.0578
{1,…,9}	26.500	9.8600
{1,…,10}	26.295	10.661
{1,…,11}	26.086	11.462
{1,…,12}	25.866	12.262
{1,…,13}	25.621	13.062
{1,…,14}	25.304	13.862

**Table 2:** The interval numbers of the statistical model.

Item	SL	SW	PL	PW
Se	[4.4,5.8]	[2.3,4.4]	[1.0,1.9]	[0.1,0.6]
Ve	[4.9,7.0]	[2.0,3.4]	[3.0,5.1]	[1.0,1.7]
Vi	[4.9,7.9]	[2.2,3.8]	[4.5,6.9]	[1.4,2.5]
Se,Ve	[4.9,5.8]	[2.3,3.4]	–	–
Se,Vi	[4.9,5.8]	[2.3,3.8]	–	–
Ve,Vi	[4.9,7.0]	[2.2,3.4]	[4.5,5.1]	[1.4,1.7]
Se,Ve,Vi	[4.9,5.8]	[2.3,3.4]	–	–

**Table 3:** BPAs based on Kang’s method, Deng extropy and final fusion result.

Item	SL	SW	PL	PW	Combined BPA
m(Se)	0.1098	0.1018	0.0625	0.1004	0.0059
m(Ve)	0.1703	0.1303	0.1839	0.2399	0.4664
m(Vi)	0.1257	0.1385	0.1819	0.3017	0.4656
m(Se,Ve)	0.1413	0.1663	0.0000	0.0000	0.0000
m(Se,Vi)	0.1413	0.1441	0.0000	0.0000	0.0000
m(Ve,Vi)	0.1703	0.1527	0.5719	0.3580	0.0620
m(Se,Ve,Vi)	0.1413	0.1663	0.0000	0.0000	0.0000
Deng extropy	5.2548	5.2806	5.1636	4.9477	

**Table 4:** The modified BPAs based on the Deng extropy and final fusion result.

Item	SL	SW	PL	PW	Combined BPA
m(Se)	0.0808	0.0730	0.0504	0.1004	0.0224
m(Ve)	0.1252	0.0934	0.1482	0.2399	0.4406
m(Vi)	0.0925	0.0993	0.1465	0.3017	0.4451
m(Se,Ve)	0.1039	0.1192	0.0000	0.0000	0.0000
m(Se,Vi)	0.1039	0.1033	0.0000	0.0000	0.0000
m(Ve,Vi)	0.1252	0.1095	0.4608	0.3580	0.0919
m(Se,Ve,Vi)	0.3684	0.4023	0.1942	0.0000	0.0000

**Table 5:** The recognition rate.

Item	Setosa	Versicolor	Virginica	Global
Kang’s method	100%	96%	84%	93.33%
Method based on Deng extropy	100%	96%	86%	94%

## Abbreviations

The following abbreviations are used in this manuscript:

BPA	Basic probability assignment
PPT	Pignistic probability transformation
SL	Sepal length in cm
SW	Sepal width in cm
PL	Petal length in cm
PW	Petal width in cm
Se	Iris Setosa
Ve	Iris Versicolour
Vi	Iris Virginica
